# High-throughput electronic property prediction of cyclic molecules with 3D-enhanced machine learning

**DOI:** 10.1039/d5sc04079e

**Published:** 2025-10-02

**Authors:** Peikun Zheng, Olexandr Isayev

**Affiliations:** a Department of Chemistry, Carnegie Mellon University Pittsburgh Pennsylvania 15213 USA olexandr@olexandrisayev.com

## Abstract

Complex organic molecules play a pivotal role in bioactive compounds and organic functional materials, yet existing molecular datasets lack structural diversity for such systems, limiting the generalizability of machine learning (ML) models. This study introduces a high-quality dataset, Ring Vault, comprising 201 546 cyclic molecules, including monocyclic, bicyclic, and tricyclic systems, spanning 11 non-metallic elements. This dataset covers a wide chemical space and provides a robust foundation for molecular property prediction. Leveraging quantum mechanical (QM) calculations on a subset (36 000 molecules), we trained three ML models (Graph Attention Network, Chemprop, and AIMNet2) to predict five key electronic properties: HOMO–LUMO gap, ionization potential (IP), electron affinity (EA), and redox potentials (*E*_ox_, *E*_red_). The fine-tuned AIMNet2 model, incorporating 3D conformational information, outperformed 2D-based models, achieving *R*^2^ values exceeding 0.95 and reducing mean absolute errors (MAEs) by over 30%. Principal component analysis (PCA) of AIMNet2 embeddings revealed intrinsic correlations between electronic properties and structural features, such as conjugation extent and functional group effects. This work establishes a robust framework for high-throughput screening and rational design of cyclic molecules, with applications spanning drug discovery, organic electronics, and energy materials. The dataset and methodology provide a foundation for exploring complex structure–property relationships and accelerating functional molecule discovery.

## Abstract 2

Ring Vault contains 201 546 cyclic molecules across 11 elements. AIMNet2 with 3D information outperformed 2D models in predicting the electronic properties of cyclic molecules.

## Graphical abstract

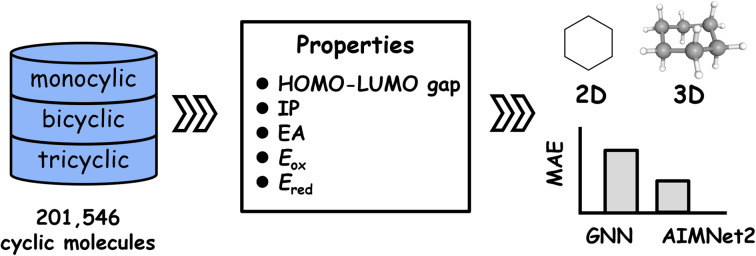

## Introduction

Ring structures form the core element in most bioactive and functional organic compounds, defining their fundamental shape and influencing flexibility or rigidity while maintaining substituents in optimal positions. These ring systems substantially affect global molecular characteristics, including polarity and hydrophobicity. Furthermore, the electronic properties of rings govern molecular reactivity, which directly impacts metabolic stability and potential toxicity.

In Computer-Assisted Drug Design (CADD) and medicinal chemistry, ring replacement strategies provide medicinal chemists with powerful tools for Structure–Activity Relationship optimization by allowing systematic exploration of how structural modifications influence biological activity against specific targets.^1^ These replacements enable fine-tuning of crucial drug-like properties such as solubility, lipophilicity, and binding selectivity, all essential for a compound's developability.^2^ Furthermore, ring replacements facilitate scaffold hopping,^3,4^ allowing researchers to explore novel chemical space while preserving core pharmacophoric features, which helps overcome patent barriers and potentially leads to compounds with improved profiles.

The importance of ring replacement in organic electronic materials and organic functional materials is also considerable. Replacing rings strategically allows materials scientists to fine-tune band gaps, frontier orbital energies (HOMO/LUMO levels), and carrier mobilities to optimize device performance. Unlike drug design, where biological activity is the primary concern, in organic electronics, the focus is on improving electronic properties, optical absorption/emission, stability, *etc.* Ring modifications can enhance π-conjugation and π-stacking interactions, which are crucial for charge transport in organic semiconductors.^5^ By replacing specific rings, researchers can adjust molecular packing in the solid state, influencing morphology and crystallinity that determine device efficiency in applications like OLEDs, organic photovoltaics, and organic field-effect transistors.^6,7^

Additionally, ring replacements provide pathways to improve material sustainability by replacing environmentally problematic structures with more benign alternatives while maintaining or enhancing performance characteristics. The systematic approach to ring replacement in functional materials can lead to breakthroughs in emerging technologies like flexible electronics, bioelectronics, and organic thermoelectrics by creating materials with precisely engineered properties.^8^

Recent advances in computational databases and machine learning (ML) frameworks have significantly expanded the tools available for molecular property prediction and accelerated the exploration of ring systems. Large-scale quantum chemical databases like QM9 (ref. 9) and QM-symex^10^ provide essential ground-state and excited-state properties for small organic molecules. The VERDE Materials Database curates over 1500 π-conjugated organic molecules with ground- and excited-state geometries and photophysical properties,^11^ while the Harvard Organic Photovoltaic dataset (HOPV15) compiles experimental photovoltaic measurements and corresponding QM calculations for organic solar-cell molecules.^12^ Furthermore, a study on photocatalytic hydrogen evolution used a 572-molecule experimental library combined with QM calculations and ML to predict activity,^13^ and a curated 25-k-molecule dataset combining DFT and TDDFT properties demonstrated the power of graph neural networks in electronic-property prediction.^14^ Recent ML advancements in redox potential prediction^15^ further demonstrate how integrating quantum-chemical descriptors with deep learning can overcome traditional DFT limitations, suggesting similar opportunities for ring system optimization.

In the realm of ring systems, the MAGIC Rings tool analyzed bioactive *versus* synthetic ring space;^16^ a library of 4 million medicinally relevant ring systems was generated based on bioactive-derived rules;^17^ the VEHICLe library catalogs 24 847 aromatic heterocycles;^18^ and a 600 k heteroaromatic scaffold database characterized by calculated structural and quantum properties supports scaffold hopping and virtual screening.^19^ The COMPAS Project's three installments (together exceeding 550 k polycyclic aromatic systems) have elucidated structure–property relationships in cata- and peri-condensed aromatic systems.^20–22^ Most recently, the RSE Atlas (16 905 monocyclic compounds) coupled with AIMNet2 machine-learning interatomic potential delivers rapid, accurate ring strain energy predictions.^23^ These datasets have significantly expanded computational data for cyclic molecular systems, providing a robust foundation for data-driven molecular property predictions.

This study presents Ring Vault, a high-quality dataset comprising over 200 000 cyclic molecules, encompassing monocyclic, bicyclic, and tricyclic structures, and incorporating 11 non-metallic elements. This dataset expands the chemical space available for molecular property prediction, enhancing both the diversity and applicability of ML models. Using this dataset, we systematically evaluated the performance of three ML models: Graph Attention Network (GAT),^24^ Chemprop,^25^ and AIMNet2.^26^ Furthermore, we employed AIMNet2 to predict the target properties of all molecules in the dataset and conducted principal component analysis (PCA) to explore the intrinsic relationship between molecular embeddings and properties. This analysis provides new theoretical insights into the relationship between molecular structure and properties, offering guidance for molecular design and high-throughput property screening.

## Method

### Dataset

**Fig. 1:**
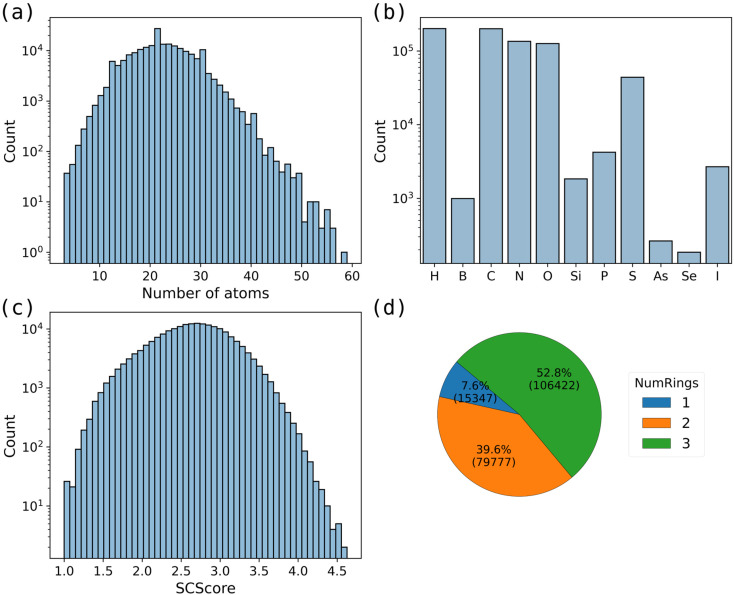
Overview of the dataset. (a) Distribution of the number of atoms per molecule. (b) Distribution of element types. (c) Distribution of SCScore values. (d) Composition of molecules with one, two, and three rings.

We have constructed the Ring Vault database containing 201 546 cyclic molecules, including 15 347 monocyclic molecules, 79 777 bicyclic molecules, and 106 422 tricyclic molecules (Fig. 1(d)). Rings are connected through fused, bridged, spiro, and diaryl-like systems. The ring sizes range from 3 to 9. Ring systems were extracted from commercially available compounds (Sigma-Aldrich,^27^ Enamine,^28^ WuXi,^29^ MolPort^30^) and literature-reported building blocks and reagents (PubChem,^31^ ChEMBL,^32^ SureChEMBL^33^). These rings represent the majority of known carbocycles and heterocycles, including an extended set of elements like S, Si, P, Se, B, and I. Fig. 1(a) and (b) presents statistical information on the distribution of atom counts and elements within the molecules in the dataset. The molecules in this dataset contain between 3 and 24 heavy atoms, with the largest molecule consisting of 59 atoms, covering a broad chemical space.

We additionally computed the Synthetic Complexity Score (SCScore)^34^ for each molecule in the dataset to assess synthetic accessibility. The distribution is presented in Fig. 1(c). The SCScore values span a broad range, demonstrating that the dataset includes both synthetically accessible molecules and those with higher synthetic complexity. Notably, 77% of the molecules have SCScore values between 1.0 and 3.0, indicating a predominance of compounds with moderate synthetic complexity.

### QM calculation

Conducting QM calculations across the entire dataset is computationally prohibitive. Therefore, we have implemented an ML approach: training a model on a small subset of the data and subsequently predicting the molecular properties for the entire dataset. This strategy is widely adopted in large-scale molecular studies, effectively reducing computational costs while maintaining high predictive accuracy.^35–37^

**Fig. 2:**
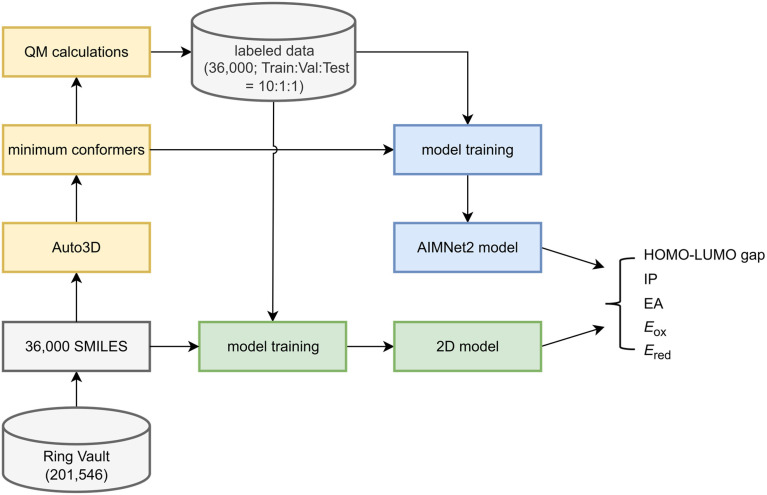
Workflow of data preparation and model training.

Fig. 2 shows the workflow of dataset construction and model training. From the constructed database, we randomly selected 30 000, 3000, and 3000 molecules as the training, validation, and test sets, respectively. To obtain reliable 3D molecular structures, we first employed the Auto3D package^38^ in combination with the pre-trained AIMNet2 (ref. 26) model to generate the lowest-energy conformations of neutral molecules from their SMILES representations. Based on these optimized conformations, we performed DFT single-point calculations for each system in three charge states (−1, 0, and +1). These calculations allowed us to determine key molecular properties of the neutral molecules, including the HOMO–LUMO gap, vertical electron affinity (EA), and vertical ionization potential (IP). Furthermore, to account for solvent effects, we carried out additional DFT single-point calculations in aqueous solution for molecules in all three charge states. These calculations enabled the estimation of oxidation and reduction potentials, which are crucial for understanding molecular redox behavior in solution.

The oxidation and reduction potentials were calculated using the following equations:1
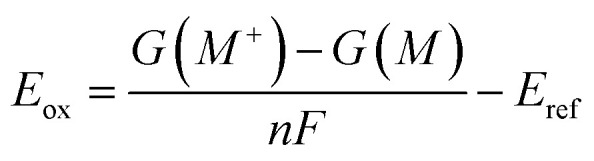
2
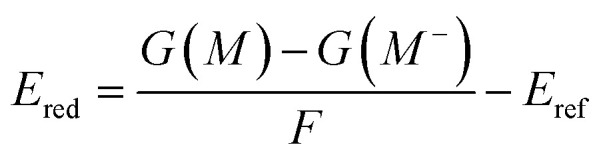
where *G*(*M*), *G*(*M*^+^), *G*(*M*^−^) represent the Gibbs free energies of the molecule in aqueous solution at the corresponding charge states. *F* is the Faraday constant, and *n* denotes the number of electrons transferred during the redox process. For all systems in this study, *n* = 1. We selected the standard hydrogen electrode (SHE) as the reference electrode, and thus the reference potential *E*_ref_ was set to 4.28 V.

All DFT calculations were performed using the ORCA 6.0.1 software^39,40^ at the *ω*B97M-D3(BJ)/def2-TZVPP^41,42^ level of theory. For solvation effects in the aqueous solution, we employed the SMD implicit solvation model in combination with Grimme's recently proposed DRACO^43^ scheme to improve the accuracy of solvation free energy predictions.

### Model training

We employed several ML algorithms to predict five key molecular properties: HOMO–LUMO gap, IP, EA, *E*_ox_, and *E*_red_. The first approach utilized a graph neural network (GNN) model. In this study, we employed the GAT model to predict the molecular properties. Specifically, the SMILES strings of molecules were first converted into molecular graph representations, where atoms were treated as nodes and chemical bonds as edges. The model architecture consisted of three GAT layers, where each layer incorporated edge features to enhance the expressiveness of the attention mechanism. After processing through the three GAT layers, global mean pooling was applied to aggregate the node-level features into a graph-level representation, creating a molecular embedding that describes the entire molecular structure. Finally, this embedding was passed through three fully connected layers, enabling iterative mapping to the predicted values for multiple molecular properties. The node features and edge features used in the GAT model are listed in Tables S1 and S2. We also used Chemprop^25^ for molecular property prediction. Chemprop is a molecular property prediction framework based on Message Passing Neural Networks (MPNN), which is widely used and serves as a benchmark for comparison.

Additionally, we fine-tuned the pre-trained AIMNet2 model^26^ using the 3D geometric information of the optimal conformations generated by Auto3D to train and predict the five molecular properties. During fine-tuning, we froze the first message-passing layer to preserve the foundational features extracted during pre-training, while allowing the subsequent layers to adjust for task-specific property mapping. The subsequent two message-passing layers generated the AIM (atoms-in-molecule) vectors, which were then processed through two fully connected layers to map these representations to the predicted values of the five molecular properties.

To clarify, two different uses of the AIMNet2 model are involved in this work. First, we employed a pretrained AIMNet2 model^26^ within the Auto3D package to efficiently generate low-energy 3D conformers from SMILES strings, enabling downstream QM calculations. This step does not involve any additional training and solely utilizes AIMNet2's learned potential for geometry optimization. Second, a separately fine-tuned AIMNet2 model was trained using our QM dataset to predict five molecular properties (HOMO–LUMO gap, IP, EA, *E*_ox_, and *E*_red_). This fine-tuning process leverages the learned 3D representations and adapts them to our specific prediction tasks.

## Results and discussion

### Model performance

**Fig. 3:**
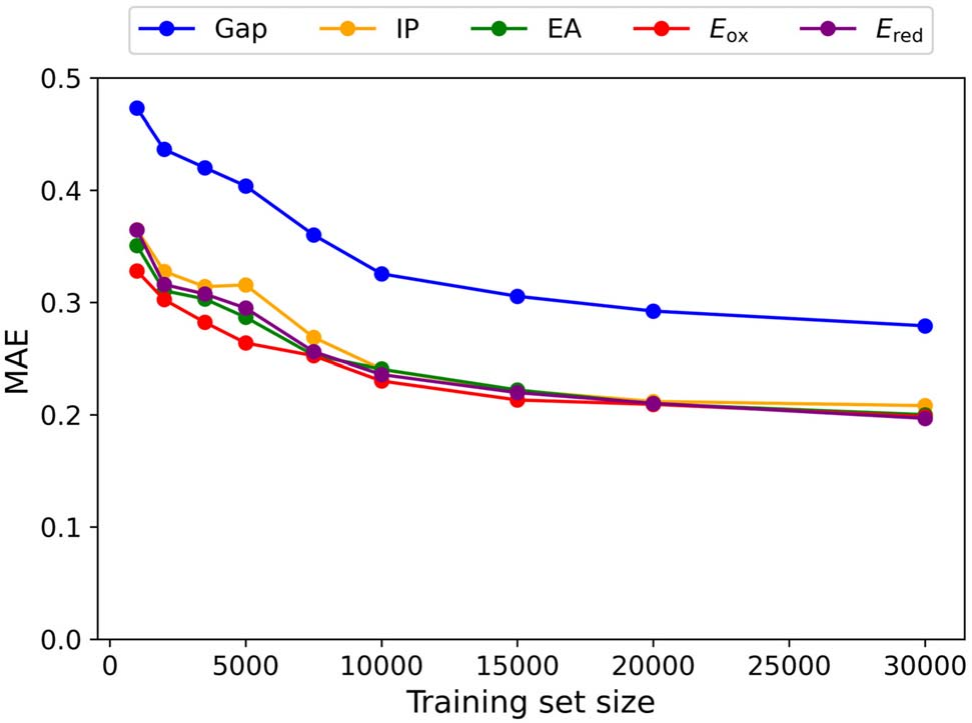
The performance of the GAT model on the test data set by using different training set sizes. The units of Gap, IP, and EA are in eV, and *E*_red_ and *E*_ox_ are in V.

Fig. 3 presents the learning curves of the GAT model. As shown, increasing the training set size from 1000 to 20 000 leads to a significant reduction in the MAE across various molecular properties, indicating enhanced predictive accuracy with more data. Further expanding the training set to 30 000 continues to reduce the MAE, albeit to a lesser extent, suggesting that the model is approaching convergence at this data scale. Therefore, we finally selected a training set size of 30 000 to balance computational cost and model accuracy.

Notably, among all predicted molecular properties, the HOMO–LUMO gap consistently exhibits the largest error, reflecting the greater difficulty in accurately predicting this property. This challenge may be attributed to the HOMO–LUMO gap's high sensitivity to electronic structure and molecular conformation.

Table 1 presents the performance of the GAT, Chemprop, and AIMNet2 models on the test set. The results indicate that all three models achieve *R*^2^ values exceeding 0.9 across five molecular properties, demonstrating their capability to accurately describe the target properties. Among them, GAT and Chemprop exhibit comparable performance, with Chemprop slightly outperforming GAT. Notably, AIMNet2 achieves the most outstanding results, with MAEs for all five properties below 0.2 and *R*^2^ values surpassing 0.95, indicating superior accuracy and reliability in molecular property prediction.

**Table 1:** The performance of different models on the test set. The units of Gap, IP, and EA are in eV, and *E*_ox_ and *E*_red_ are in V

	GAT			Chemprop			AIMNet2		
	MAE	RMSE	*R* ^2^	MAE	RMSE	*R* ^2^	MAE	RMSE	*R* ^2^
Gap	0.279	0.422	0.931	0.273	0.408	0.935	0.163	0.242	0.977
IP	0.208	0.301	0.925	0.196	0.277	0.936	0.131	0.194	0.969
EA	0.200	0.306	0.948	0.195	0.301	0.950	0.111	0.169	0.984
*E*_ox_	0.199	0.284	0.914	0.191	0.269	0.923	0.128	0.189	0.962
*E*_red_	0.197	0.303	0.944	0.200	0.301	0.944	0.122	0.180	0.980

**Fig. 4:**
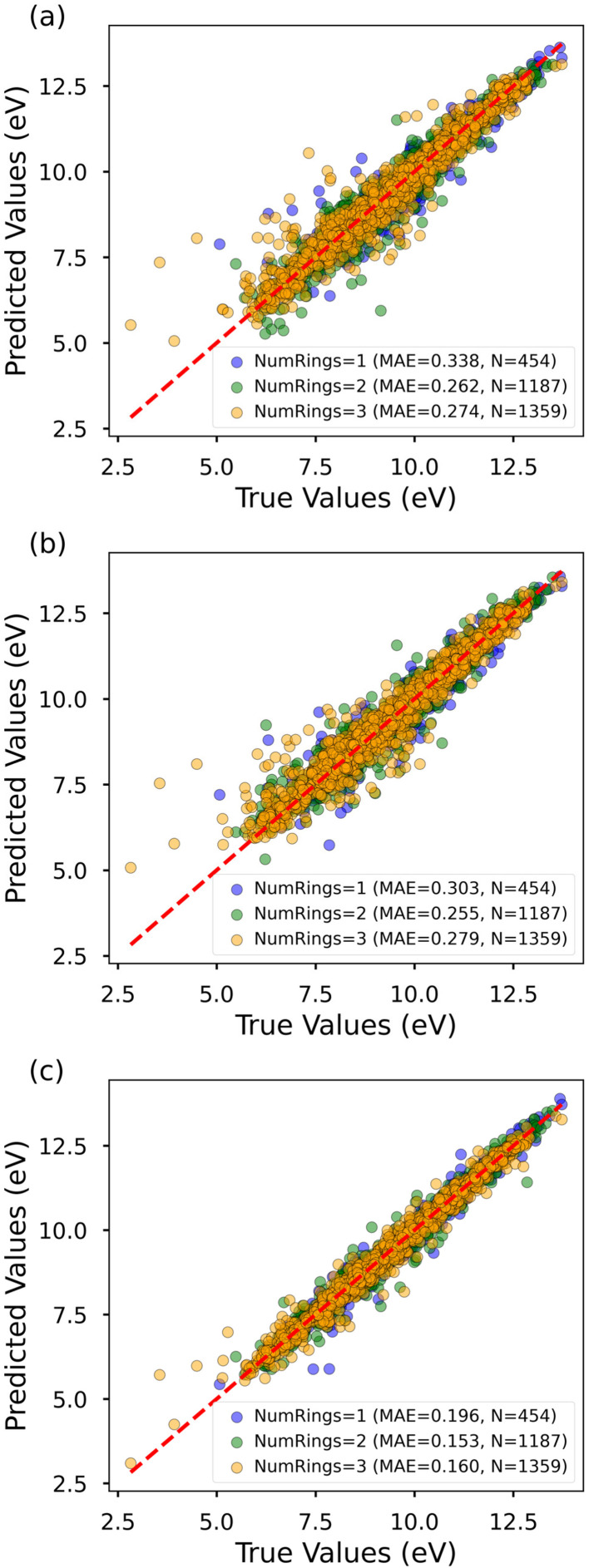
Performance of GAT (a), Chemprop (b), and AIMNet2 (c) model for the HOMO–LUMO gap on the test data set.

Given that the prediction error for the HOMO–LUMO gap is relatively larger among the five properties, we conducted a detailed analysis of molecules with different ring sizes, as depicted in Fig. 4 (performances for other properties are provided in the SI, Fig. S1–S4). The findings reveal that, across all models, monocyclic molecules exhibit the highest MAE, primarily due to their limited representation in the dataset. For instance, among the 30 000 training samples, the counts for monocyclic, bicyclic, and tricyclic molecules are 4,526, 12 018, and 13 456, respectively, indicating a notable underrepresentation of monocyclic samples. While augmenting the training data for monocyclic molecules could further enhance prediction accuracy, the AIMNet2 model has already achieved an MAE of 0.163 eV for this property. This level of accuracy allows the model to reliably capture structure–property trends across diverse molecular scaffolds, making it suitable for high-throughput screening and early-stage molecular design. However, for applications requiring very fine energetic discrimination (*e.g.*, <0.05 eV) or direct comparison to experimental measurements, additional high-level validation remains essential.

As shown in Fig. 4(c), a small number of molecules shows notably large prediction errors. To better understand the underlying reasons for these outliers, we closely examined the 10 molecules with the largest absolute errors in the test set. According to the structures shown in Fig. S5, these molecules share several unusual structural characteristics, including highly strained ring systems, irregular conjugation patterns, silicon-containing heterocycles, and cumulated dienes. These structural motifs are rarely observed in the training set and are likely underrepresented in the model's learned chemical space, which limits its ability to generalize to such cases.

To quantify their uniqueness, we computed the structural similarity between each high-error molecule and the full dataset using Morgan fingerprints (radius = 3) and Tanimoto similarity. The majority of similarity scores were below 0.5, suggesting that these molecules have few close analogs in the training data and likely fall in underexplored regions of chemical space. These findings underscore the critical importance of training set diversity and coverage, especially when developing models intended to generalize across a broad range of molecular structures.

### Predicting the full dataset with the AIMNet2 model

The above results have demonstrated that the AIMNet2 model, which incorporates 3D molecular structural information, outperforms the GAT and Chemprop models that rely solely on 2D molecular topology in predicting molecular properties. Therefore, we utilized AIMNet2 to predict the target properties for all molecules in the dataset and further analyzed the distribution characteristics and interrelationships of these properties.

**Fig. 5:**
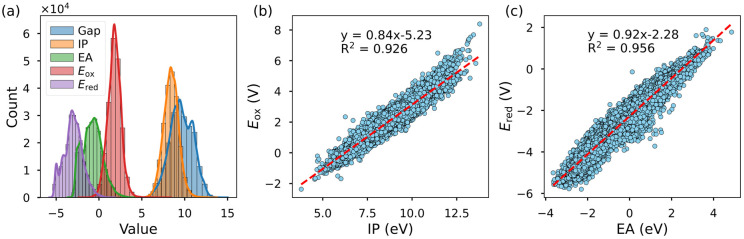
(a) Histogram of the distribution of five properties predicted by the AIMNet2 model on the entire dataset. (b) Correlation between IP and *E*_ox_. (c) Correlation between EA and *E*_red_.

Fig. 5(a) illustrates the distribution of five molecular properties. The HOMO–LUMO gap ranges from 3.05 to 15.02 eV, IP spans from 3.79 to 13.78 eV, EA varies between −3.64 and 4.86 eV, *E*_ox_ ranges from −2.37 to 8.39 eV, and *E*_red_ spans from −5.80 to 1.89 eV. These distributions highlight the dataset's broad molecular diversity, covering systems with a wide range of redox characteristics, from strong oxidants to strong reductants.

In Fig. 5(b) and (c), we analyzed the relationships between *E*_ox_ and IP, as well as *E*_red_ and EA, respectively. The results show that both property pairs exhibit *R*^2^ values exceeding 0.9, indicating a strong linear correlation. Since IP is commonly approximated by the HOMO energy, and EA can be derived from the LUMO energy, some approaches directly predict redox potentials based on molecular orbital energies.^15^ This strong correlation not only validates the predictive reliability of AIMNet2 but also provides theoretical insight into the relationship between redox properties and molecular orbital energies.

**Fig. 6:**
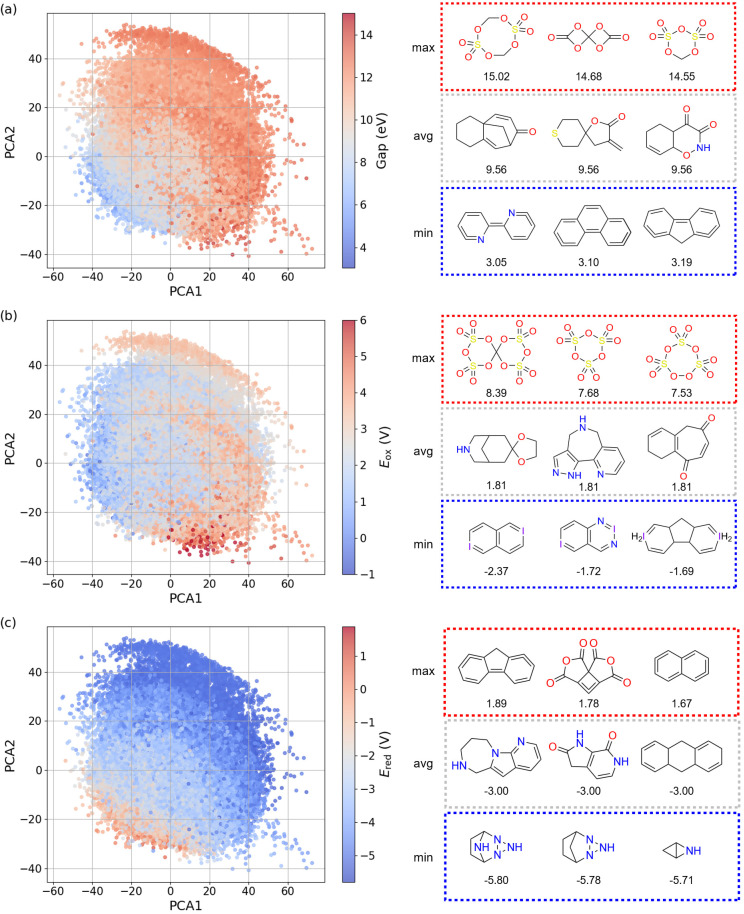
PCA projection of the AIMNet2 molecular embeddings. (a) Points color-coded by HOMO–LUMO gap (eV). (b) Points color-coded by *E*_ox_ (V). (c) Points color-coded by *E*_red_ (V). The three molecules with the highest, lowest, and closest-to-average values for each property are highlighted on the right panel.

In Fig. 6, we performed PCA on the molecular embeddings learned by AIMNet2 to reduce their dimensionality. Given the strong linear correlations between IP and *E*_ox_, as well as between EA and *E*_red_, we present only the distributions of Gap, *E*_ox_, and *E*_red_ in the figure. Along the PCA1 axis, the HOMO–LUMO gap exhibits a distinct gradient trend: molecules with lower gap values (blue) are predominantly located on the left, while those with higher gap values (red) are concentrated on the right. This trend suggests that PCA1 is closely related to key electronic structural features of the molecules, such as the degree of conjugation or the presence of specific functional groups. Analyzing the representative molecular structures, we find that low-gap compounds typically possess extended conjugation, whereas those containing anhydride or sulfonic anhydride functional groups tend to exhibit higher gap values. This observation is consistent with molecular orbital theory, which shows that larger conjugated systems generally result in a reduced HOMO–LUMO gap, thereby enhancing the molecule's optical and electrochemical activity.

The distribution of *E*_ox_ follows a trend similar to that of the HOMO–LUMO gap but exhibits a more gradual color gradient transition. This suggests that while *E*_ox_ is also influenced by molecular structure, additional factors may contribute to its variability. Notably, compounds containing iodine within conjugated rings tend to exhibit lower *E*_ox_ values, whereas sulfate anhydrides correspond to higher *E*_ox_ values. This trend can be attributed to iodine's electron-donating ability, which facilitates electron loss and lowers the oxidation potential. In contrast, the sulfate anhydride group is highly electron-withdrawing, making electron removal more difficult and resulting in a higher oxidation potential.

The distribution trend of *E*_red_, on the other hand, is inversely related to that of the HOMO–LUMO gap, indicating that molecules with smaller gap values are generally more prone to reduction (*i.e.*, they exhibit more negative *E*_red_ values). Structural analysis reveals that cyclic amidine compounds often exhibit lower *E*_red_ values, suggesting that these compounds readily accept electrons and thus display stronger reducing properties.

These findings demonstrate that the molecular embeddings learned by AIMNet2 effectively capture the intrinsic relationships between different molecular properties and accurately reflect the impact of molecular structure on properties. This further validates the model's reliability and physical interpretability in predicting molecular electronic properties. However, we acknowledge that a more detailed analysis incorporating quantitative physical organic chemistry descriptors (such as Hammett parameters, aromaticity indices, and orbital coefficients) could provide deeper mechanistic insights. Such comprehensive structure–property analysis represents a valuable direction for future work, particularly once the model is applied to specific chemical design problems where understanding these relationships becomes critical for rational molecular optimization.

Although aqueous solution was employed as the solvent environment for redox potential calculations in this study, we acknowledge the intrinsic limitations of quantum chemical predictions in reproducing experimental redox potentials. Implicit solvation models, while computationally efficient, are unable to fully account for specific solute–solvent interactions, hydrogen-bonding networks, and ionic strength effects that critically influence redox behavior in solution.^44–46^ Moreover, proton-coupled electron transfer (PCET) processes often govern redox reactivity in aqueous environments, and accurate modeling of such processes typically requires the explicit inclusion of protons and solvent molecules.^47–49^

To mitigate some of these limitations, we adopted the DRACO model. As benchmarked by Grimme and co-workers, the SMD with DRACO(EEQ) approach significantly improves the accuracy of solvation free energy predictions, particularly for charged solutes, yielding a mean absolute deviation (MAD) of only 3.2 kcal mol^−1^ for the S30L dataset.^43^ This enhancement in solvation modeling contributes to more reliable estimation of redox potentials, albeit still within the broader constraints of implicit solvation.

Overall, while the redox potentials reported here may not quantitatively match experimental measurements, they serve as valuable proxies for relative comparisons and structure–property trend analysis across large molecular spaces.

### Tuning molecular properties

**Fig. 7:**
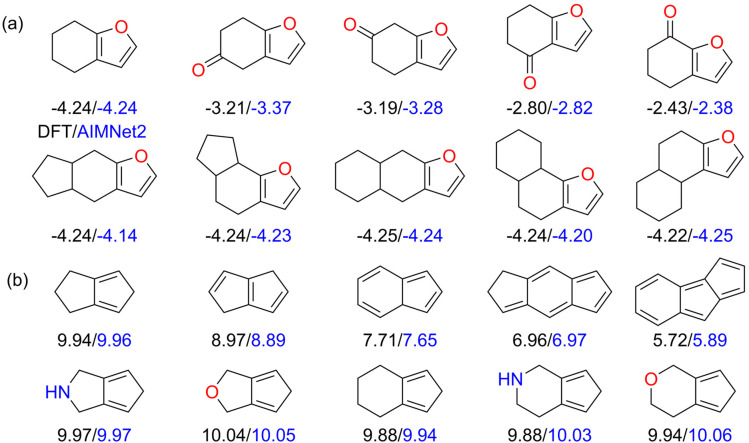
Effect of molecular structural modifications on (a) *E*_red_ (V) and (b) the HOMO–LUMO gap (eV). Black values correspond to DFT calculations, while blue values represent AIMNet2 predictions.

Fig. 7 illustrates the significant impact of molecular structural design on the *E*_red_ and the HOMO–LUMO gap. As shown in the Fig. 7, the AIMNet2 predictions for both *E*_red_ and the HOMO–LUMO gap closely match the DFT results, with most deviations falling below 0.1 eV. In Fig. 7(a), the *E*_red_ of the molecules in the first row predicted by AIMNet2 gradually increases from −4.25 eV to −2.38 eV, indicating a marked enhancement in their electron-accepting ability. This trend can be attributed to the introduction of electron-withdrawing carbonyl groups (–C=O) above the ring, which effectively stabilizes the reduced state of the molecule, thus lowering the *E*_red_. It is important to note that the position of the carbonyl group within the molecule also affects the reduction potential: when the carbonyl group is closer to the furan ring, *E*_red_ decreases more significantly. In contrast, the molecules in the second row of Fig. 7(a) show only slight fluctuations in *E*_red_, ranging from −4.25 to −4.14 eV. Despite differences in the configurations of the side-chain rings (such as ring size and connectivity), the preservation of the positioning of the furan ring results in only minor changes in *E*_red_. This finding is of considerable importance for the design of molecules that require consistent redox properties, such as stable electrode materials or antioxidants.

Fig. 7(b) shows how the HOMO–LUMO gap evolves with structural changes. As the size and number of rings increase, the degree of π-electron delocalization improves, leading to enhanced molecular conjugation and a significant reduction in the HOMO–LUMO gap. This result is consistent with classical Hückel molecular orbital theory. For the molecules in the second row of Fig. 7(b), modifications such as substituting carbon atoms with nitrogen or oxygen, or expanding the size of the side-chain rings, result in relatively smaller variations in the HOMO–LUMO gap.

In summary, Fig. 7 shows the importance of structural modifications in tuning molecular properties. By rationally adjusting molecular structures, such as changing ring sizes, modulating the degree of conjugation, introducing specific functional groups, or substituting heteroatoms, one can achieve targeted control over desired properties at the molecular design level. Leveraging the AIMNet2 model proposed in this work, it becomes feasible to efficiently screen large libraries of candidate molecules, thereby enabling the rapid discovery of novel functional molecules that meet specific property requirements and accelerating the process of molecular design and discovery.

## Conclusions and outlook

This study proposes an ML-based approach for efficiently predicting key electronic properties of cyclic molecular systems. We constructed a comprehensive database covering monocyclic, bicyclic, and tricyclic molecules, generated low-energy conformations using Auto3D, and obtained reference data through DFT calculations. Based on this dataset, we employed three models—GAT, Chemprop, and AIMNet2—to predict five critical electronic properties, including the HOMO–LUMO gap, IP, EA, *E*_ox_, and *E*_red_. The results show that while all models achieve high predictive accuracy, the ring-specific refinement AIMNet2 model, which incorporates 3D conformational information, outperformed the others. Specifically, AIMNet2 significantly outperforms the 2D-based GAT and Chemprop models, achieving *R*^2^ values exceeding 0.95 and reducing the MAE by more than 30% on the test set.

The long-standing debate in chemistry literature regarding 2D *versus* 3D molecular representations reveals a nuanced landscape of trade-offs and applications.^50–52^ The fundamental distinction lies in how molecular information is encoded: graphs serve as formally 2D data structures that lack spatial relationships between elements. In contrast, 3D representations capture spatial coordinates, conformational ensembles, and geometric relationships. The conformation of a molecule typically incorporates the atomic 3D coordinates of that molecule.

Recent studies indicate that the effectiveness of 2D *versus* 3D representations depends heavily on the specific properties involved. As we show in the present study, utilizing 3D information often leads to significant advantages for quantum mechanical and electronic properties. The significance of geometric details is particularly pronounced for conformational properties, as molecular geometry is essential for determining molecular characteristics.^53^ It is important to note that this principle does not apply universally to all molecular properties. Interestingly, the advantages of using molecular geometries were not recognized in the prediction of liquid-phase or critical properties. In fact, 2D models achieved comparable or even superior performance compared to 3D models in both inter- and extrapolative evaluations.^54^

Further analysis of ML models revealed distinct distribution patterns of molecular properties in the embedding space. Specifically, the HOMO–LUMO gap and *E*_ox_ exhibited clear gradient variations, while the *E*_red_ and EA displayed an inverse correlation. These trends are closely linked to the degree of molecular conjugation and the electron-donating or withdrawing capabilities of functional groups. Additionally, PCA indicates that key structural features of molecules can be effectively captured within a low-dimensional embedding space. This not only enhances the interpretability of the models but also provides valuable insights into the intrinsic relationships between molecular structure and electronic properties.

Looking ahead, several promising research directions emerge. First, expanding our approach to more complex polycyclic systems and incorporating additional heteroatoms could further broaden the applicability of our models. Second, integrating our framework with active learning techniques would enable a more efficient exploration of chemical space, potentially uncovering novel molecules with tailored properties. Third, extending our methodology to predict additional properties relevant to specific applications, such as optical properties or thermal stability, would enhance its utility for targeted material design. Although aqueous solution was employed as the solvent environment for redox potential calculations in this study, we acknowledge that organic solvents (*e.g.*, acetonitrile, dimethylformamide) are often more representative of practical applications, especially in organic electronics and non-aqueous electrochemical systems. Future work could readily extend property predictions to specific organic solvents by generating training data using the corresponding implicit solvation model for the target solvent, while leveraging the same model architecture and fine-tuning strategy. Finally, developing interpretable ML models that provide deeper insights into the fundamental QM principles governing electronic properties would bridge the gap between data-driven predictions and theoretical understanding.

The framework established in this study provides not only a computational tool for high-throughput screening but also a foundation for rational molecular design in diverse fields, including organic electronics, energy storage, and pharmaceutical development. By continuing to refine these approaches, we anticipate accelerating the discovery of functional molecules with optimized properties for addressing contemporary technological challenges.

## Author contributions

P. Z.: performed calculations, visualization, data curation. O. I.: supervision, conceptualization. All authors contributed to the writing of the manuscript and analysis of the results.

## Conflicts of interest

The authors declare no competing interests.

## Supplementary Material

SC-OLF-D5SC04079E-s001

## Data Availability

The dataset and trained AIMNet2 model for ring systems are available at https://github.com/pkzheng94/ring.git. Supplementary information: details of model hyperparameters, parity plots of all property predictions, and prediction outliers. See DOI: https://doi.org/10.1039/d5sc04079e.
