# Supplementary material for: High-throughput electronic property prediction of cyclic molecules with 3D-enhanced machine learning
Source: Chem Sci. 2025 Oct 2;16(43):20553–63. doi: 10.1039/d5sc04079e (PMC12502961; doi:10.1039/d5sc04079e)
Supplement: SC-016-D5SC04079E-s001 [file SC-016-D5SC04079E-s001.pdf]

Supplementary Information (SI) for Chemical Science.
This journal is © The Royal Society of Chemistry 2025

# Supporting Information:

# High-Throughput Electronic Property Prediction of Cyclic Molecules with 3D-Enhanced Machine Learning

Peikun Zheng, Olexandr Isayev*

[1]*Department of Chemistry, Carnegie Mellon University, Pittsburgh, Pennsylvania 15213, United States*

*E-mail: olexandr@olexandrisayev.com*

**Table S1.** The description of the used node features.

| feature | description |
|---|---|
| atomic number | A one-hot vector representing the atom type: B, C, N, O, Si, P, S, As, Se, I |
| degree | A one-hot vector representing the degree of atom |
| charge | A one-hot vector representing the formal charge of the atom |
| hybridization | A one-hot vector indicating the hybridization state of the atom |
| in ring | A binary value indicating whether the atom is part of a ring structure |
| aromatic | A binary value indicating whether the atom is aromatic |
| atomic mass | A scaled value representing the atomic mass of the atom |
| vdW radius | A scaled value representing the vdW radius of the atom |
| covalent radius | A scaled value representing the covalent radius of the atom |
| chirality | A one-hot vector indicating the chirality of the atom |
| number of hydrogens | A one-hot vector representing the number of hydrogen atoms directly bonded to the atom |

**Table S2.** The description of the used bond features.

| feature | description |
|---|---|
| bond type | A one-hot vector representing the bond type |
| conjugated | A binary value indicating whether the bond is conjugated |
| in ring | A binary value indicating whether the bond is part of a ring structure |
| stereo | A one-hot vector representing the stereochemistry of the bond |

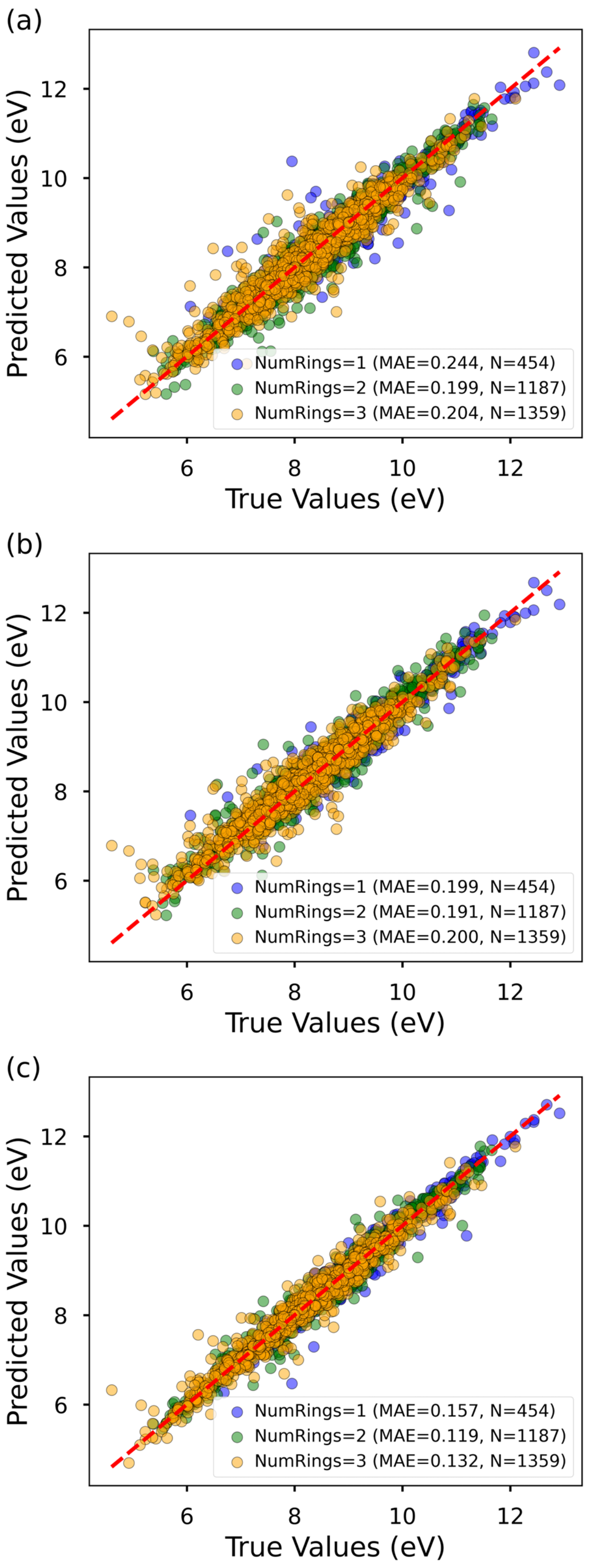


**Figure S1.** Performance of GAT (a), Chemprop (b) and AIMNet2 (c) model for the IP on the test data set.

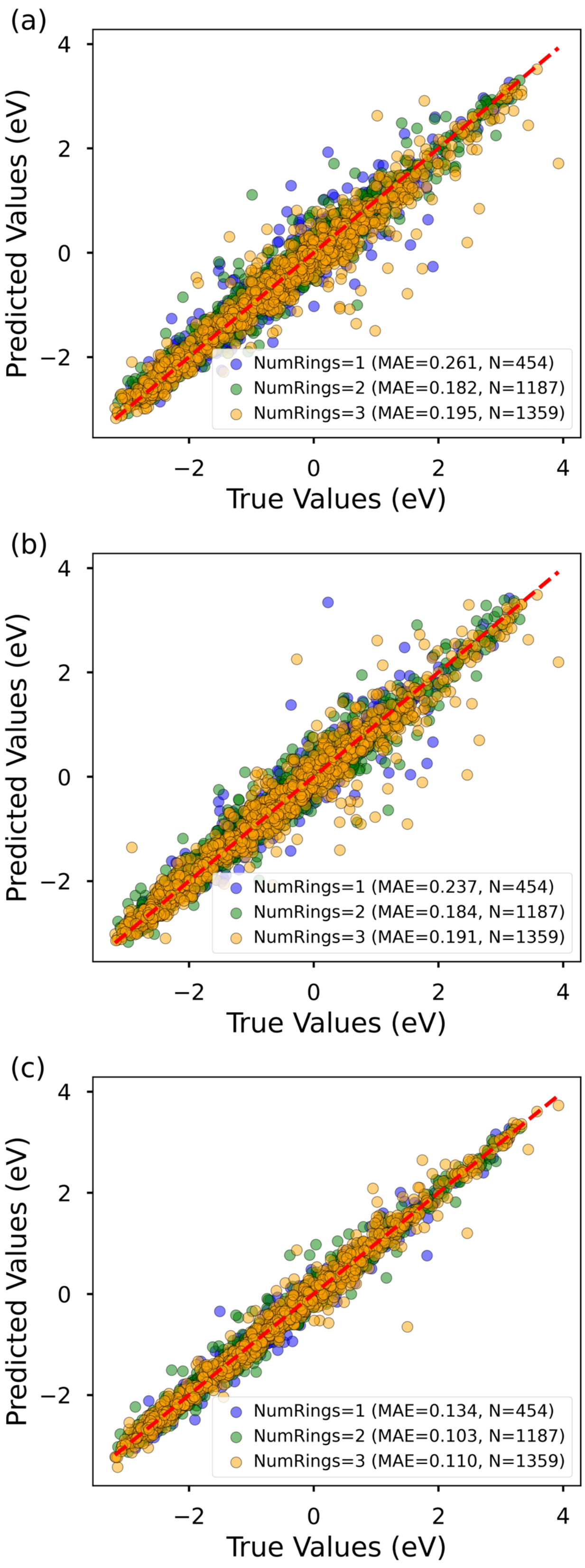


**Figure S2.** Performance of GAT (a), Chemprop (b) and AIMNet2 (c) model for the EA on the test data set.

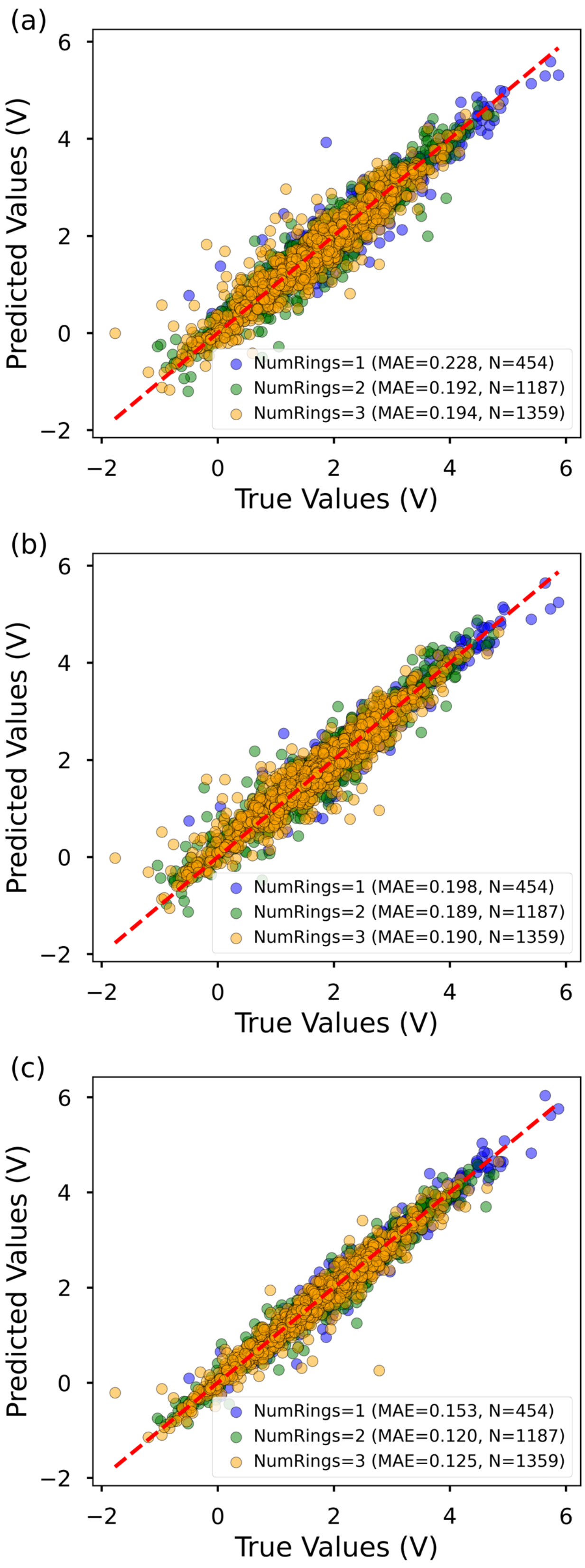


**Figure S3.** Performance of GAT (a), Chemprop (b) and AIMNet2 (c) model for the $E_{ox}$ on the test data set.

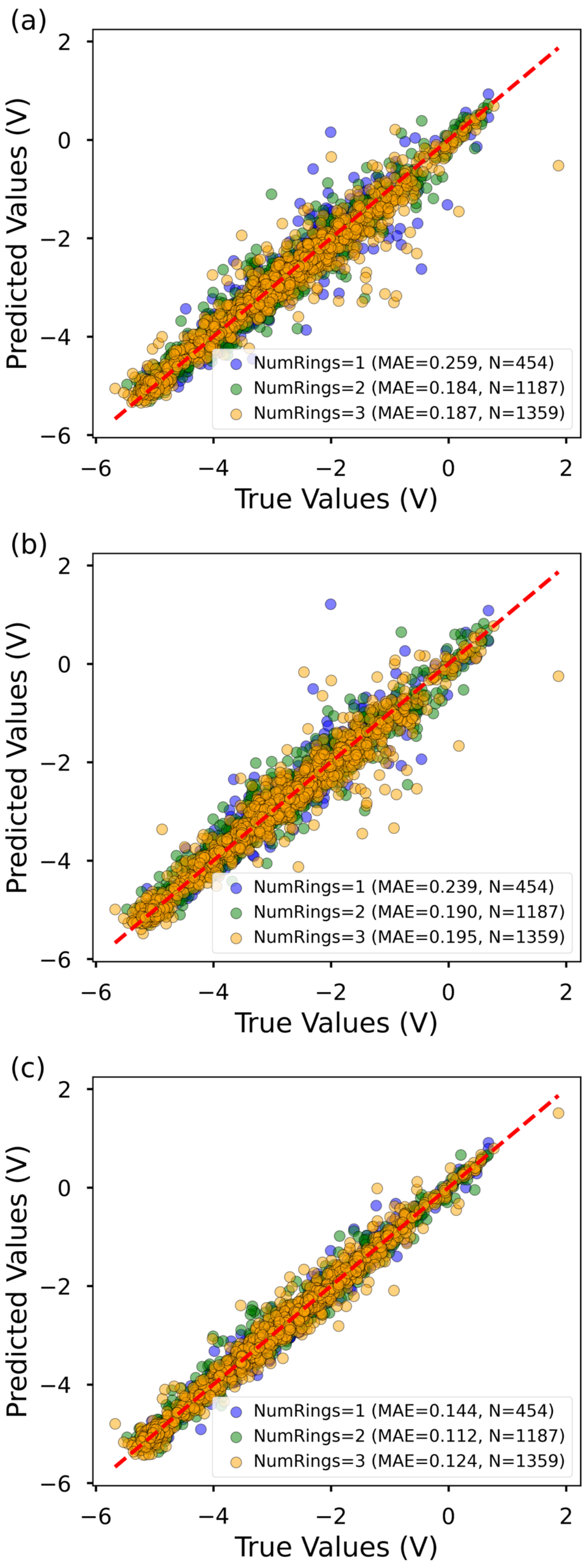


**Figure S4.** Performance of GAT (a), Chemprop (b) and AIMNet2 (c) model for the $E_{red}$ on the test data set.

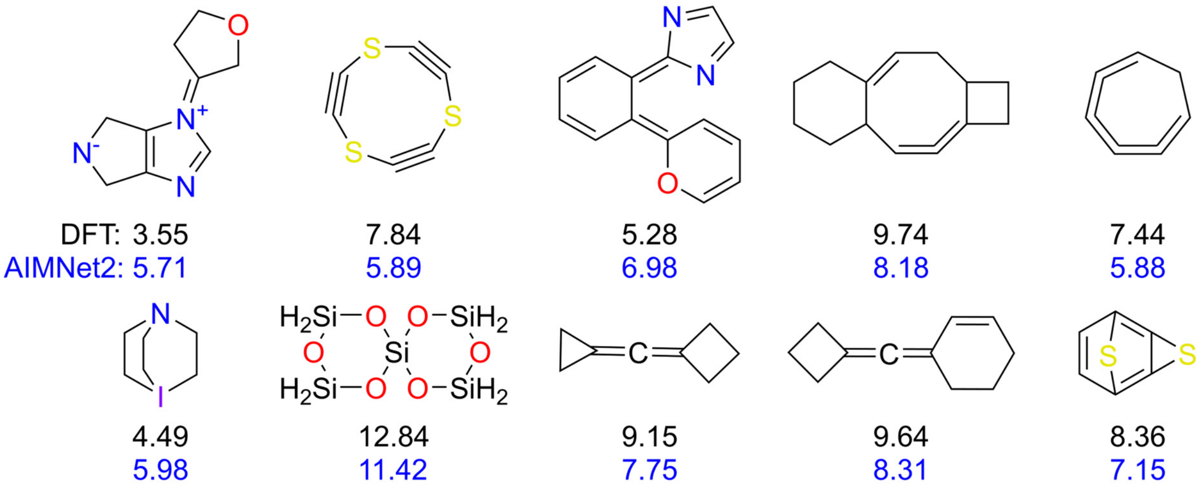


**Figure S5.** Molecular structures of the top 10 test set molecules with the highest HOMO-LUMO gap (in eV) prediction errors. Black values correspond to DFT calculations, while blue values represent AIMNet2 predictions.
